# An Empirical Study of Univariate and Genetic Algorithm-Based Feature Selection in Binary Classification with Microarray Data

**Published:** 2007-02-23

**Authors:** Michael Lecocke, Kenneth Hess

**Affiliations:** 1 Department of Mathematics, St. Mary’s University, San Antonio, Texas 78228, U.S.A; 2 Department of Biostatistics and Applied Mathematics, UT MD Anderson Cancer Center, Houston, Texas 77030, U.S.A; 3 Department of Epidemiology and Biostatistics, University of Texas Health Science Center, San Antonio, Texas 78229, U.S.A

**Keywords:** cross-validation, feature selection, supervised-learning, genetic algorithm

## Abstract

**Background:**

We consider both univariate- and multivariate-based feature selection for the problem of binary classification with microarray data. The idea is to determine whether the more sophisticated multivariate approach leads to better misclassification error rates because of the potential to consider jointly significant subsets of genes (but without overfitting the data).

**Methods:**

We present an empirical study in which 10-fold cross-validation is applied externally to both a univariate-based and two multivariate- (genetic algorithm (GA)-) based feature selection processes. These procedures are applied with respect to three supervised learning algorithms and six published two-class microarray datasets.

**Results:**

Considering all datasets, and learning algorithms, the average 10-fold external cross-validation error rates for the univariate-, single-stage GA-, and two-stage GA-based processes are 14.2%, 14.6%, and 14.2%, respectively. We also find that the optimism bias estimates from the GA analyses were half that of the univariate approach, but the selection bias estimates from the GA analyses were 2.5 times that of the univariate results.

**Conclusions:**

We find that the 10-fold external cross-validation misclassification error rates were very comparable. Further, we find that a two-stage GA approach did not demonstrate a significant advantage over a 1-stage approach. We also find that the univariate approach had higher optimism bias and lower selection bias compared to both GA approaches.

## Background

### Motivation

DNA microarray technology has greatly influenced the realms of biomedical research, with the hopes of significantly impacting the diagnosis and treatment of diseases. Microarrays have the ability to measure the expression levels of thousands of genes simultaneously. They measure how much a given type of messenger RNA (mRNA) is present in a tissue sample at a given moment. The wealth of gene expression data that has become available for microarray data analysis has introduced a number of statistical questions to tackle. Some questions are targeted towards various preprocessing stages of a microarray experiment such as RNA hybridization to arrays, image processing, and normalization, while others are geared towards assessing differential expression and identifying profiles for classification and prediction. Within the framework of tumor classification, the types of goals that have been explored include discovering or identifying previously unknown tumor classes, classifying tumors into previously known classes, and identifying “marker genes” that characterize various tumor classes.

In standard discrimination problems, the number of training observations *N* is usually much larger than the number of feature variables *p*. However, in the context of microarrays, the number of tissue samples *N* is usually between 10 and 100, significantly smaller than the thousands of genes considered in a typical microarray analysis. This presents a number of problems to a prediction rule in a discriminant analysis setting. The prediction rule may not even be able to be formed using all *p* variables, as is the case with Fisher’s linear discriminant analysis (Ambriose and McLachlan, 2002). Further, even if all the variables could be taken into account in forming the prediction rule, some of them may possess minimal (individual) discriminatory power, potentially inhibiting the performance of the prediction rule when applied to new (unclassified) tumors.

Ultimately, with a collection of genes that have high discriminatory power, an effective prediction rule can be developed based on these genes and used to allocate subsequent unclassified tissue samples as one of two classes such as cancer and normal, or perhaps as one of two subtypes of a particular cancer. Discovery of key genes needed for accurate prediction could pave the way to better understand class differences at the molecular level, which could hopefully provide more information about how to select important biomarkers to be used in the development of clinical trials for predicting outcome and various forms of treatment.

### Supervised Learning

Gene expression data for *p* genes over each of *N* mRNA samples can be expressed as an *N x p* matrix *X* = (*x_ij_* ), *i* = 1, ..., *N* and *j* = 1, ..., *p*). Each value *x_ij_* corresponds to the expression level for gene *j* in sample *i*. Each sample would have associated with it a gene expression profile *x_i_* = (*x_i_*_1_, *x_i_*_2_, . . . , *x_ip_*) ε *R*^p^, along with its class designation *yi*. This variable serves as the response, or dependent variable, and can take one of two predefined values from {0, 1}. Using the observed measurements *X*, a classifier for two classes is thus a mapping *G : R^p^* → {0, 1}, where *G*(*x*) denotes the predicted class, *y_pred_* = *c, c* ε{0, 1}, for a sample with feature vector *x*.

The samples already known to belong to certain classes, *L* = {(*x*_1_, *y*_1_), (*x*_2_, *y*_2_), . . . , (*x_nL_*, *y_nL_*)}, constitute the training (or learning) set. The training set is used to construct a classifier, which is then used to predict the classes of an independent set of samples (the test set *T* = {*x*_1_, *x*_2_, . . . , *x_nT_*}). This way, the class *y_i pred_*, (*i* = 1, 2, ..., *nT* ) predictions for each test set expression profile *x_i_* can be made. Of course, with the true classes *y_i_*, (*i* = 1, 2, ..., *nT*) of the test set known, a misclassification error rate (MER) can then be computed.

### Feature Subset Selection

In general, feature (variable) selection is an important aspect of classification problems, since the features selected are used to build the classifier. Careful consideration should be given to the problem of feature subset selection with high-dimensional data. With respect to microarray data, this of course amounts to reducing the number of genes used to construct a prediction rule for a given learning algorithm. There are several reasons for performing feature reduction. Whereas two variables could be considered good predictors individually, there could be little to gain by combining the two variables together in a feature vector. It has been reported that as model complexity is increased with more genes added to a given model, the proportion of training samples (tissues) misclassified may decrease, but the misclassification rate of new samples (generalization error) would eventually begin to increase; this latter effect being the product of overfitting the model with the training data (McLachlan, 1992; Theodoridis, 1999; Hastie et al. 2001; Xiong, 2001; Xing, 2002). Further, if another technology will be used to implement the gene classifier in practice (e.g. to develop diagnostic assays for selected subsets of genes), the cost incurred is often a function of the number of genes. Finally, there is the obvious issue of increased computational cost and complexity as more and more features are included in a model.

### Univariate and Multivariate Feature Subset Selection

Feature selection can be performed in a univariate or multivariate fashion (i.e. performing feature selection based on a single gene at a time, or considering subsets of genes at a time). The most common approach to univariate feature selection, which has been used extensively for years in the context of binary classification problems, is the simple *t*-test used to measure the degree of gene expression difference between two types of samples (groups). The basic idea with univariate feature selection is to create a ranked-list of genes based on their individual scores (e.g. properly adjusted *p*-values). With respect to multivariate feature selection, the basic idea is to consider the predictive ability of groups of genes together. This study implements a particular type of multivariate feature selection technique–an evolutionary algorithm known as the genetic algorithm (GA), the basics of which are introduced in the following section.

### Genetic Algorithm

The basic premise of a GA is to apply the principles of natural evolution and selection as a means of determining an optimal solution to a feature subset selection problem. Genetic algorithms begin with an initial population of randomly chosen candidate solutions (or chromosomes, or individuals) to use for classification of a given sample as one of two types. Each solution is of a pre-specified length (i.e. composed of a particular number of features, or genes). Through several basic processes, these solutions then evolve toward better solutions (where for this study, the notion of “better” corresponds to more accurate discriminatory power between two groups of samples). One of the basic processes used is that of *selection*, which parallels the “survival of the fittest” notion of evolution. In each generation, the fitness of every solution in the population is evaluated, multiple individuals are stochastically selected from the current population (based on their fitness). The “most fit” members of the population survive to be candidates for the next generation of solutions (next population), whereas the “least fit” members are dismissed. The fitness function can be any of a number of metrics or supervised learning algorithms (for this study, simple mahalanobis distance is used within the GA). The process of selection is also what drives the selection of the initial population of solutions from the original gene pool. Selected solutions then become part of the process of *crossover*, which parallels the crossover of DNA strands that occurs during reproduction in living organisms. This genetic operation seeks to combine elements of existing solutions together and form new and potentially better solutions (“offspring”) with some features from each “parent.” These new solutions comprise the subsequent generation (population) of solutions. The third basic process of a GA is mutation, which is used to maintain genetic diversity of solutions from one generation to those of the next generation. This process parallels the role of biological mutation within an organism’s DNA in natural evolution. The mutation operation in a GA usually involves a very small probability that an arbitrary gene from some newly-created solutions will be replaced with a different gene from the original gene pool. Ultimately, through these three genetic operations, optimal (or as close to optimal as possible) candidate solutions are generated after evolving through a pre-specified number of generations. More details on the actual parameterization of the GA used in this study, including a schematic of the basic GA process, are provided in Methods.

### Assessing the Performance of a Prediction Rule: Cross-validation

One approach to estimate the error rate of a prediction rule would be to apply the rule to a “held-out” test set randomly selected from among the training set samples. As an alternative to the “hold-out” approach, cross-validation (CV) is very often used, especially when one does not have the luxury of withholding part of a dataset as an independent test set and possibly even another part as a validation set (usually the case with microarray data). Further, the repeatability of results on new data can be assessed with this approach. In general, all CV approaches can fall under the “*K*-fold CV” heading. Here, the training set of samples is divided into *K* non-overlapping subsets of (roughly) the same size. One of the *K* subsets is “held-out” for testing, the prediction rule is trained on the remaining *K* − 1 subsets, and an estimate of the error rate can then be obtained from applying each stage’s prediction rule to its corresponding test set. This process repeats *K* times, such that each subset is treated once as the test set, and the average of the resulting *K* error rate estimates forms the *K*-fold CV error rate. The whole *K*-fold CV process could be repeated multiple times, using different partitions of the data each run and averaging the results, to obtain more reliable estimates. Leave-one-out CV (LOO CV) represents the extreme case of *K*-fold CV. This type of CV occurs when *K* = *N*, the number of samples. In this case, each sample serves as its own test set. Although it is nearly unbiased, it is generally highly variable and requires considerable computation time. At the expense of increased computation cost, repeated-(10-) run CV has been recommended as the procedure of choice for assessing predictive accuracy of the classification of microarray data (Kohavi, 1995; Braga-Neto and Dougherty, 2004; Molinaro et al. 2005A). With microarray classification problems, the practice has generally been to perform CV only on the classifier construction process, not taking into account feature selection. The feature selection process is applied to the entire set of data. This approach to CV is referred to as “internal” cross-validation (McLachlan, 1992; Ambroise and McLachlan, 2002; Dudoit and Fridlyand, 2003).

Although the intention of CV is to provide accurate estimates of classification error rates, using CV in this manner means that any inference would be made with respect to the classifier building process only. Leaving out feature selection from the cross-validation process will inevitably lead to selection bias, as the feature selection would not be based on the particular training set for each CV run. Hence, overly optimistic error rates would be obtained. To prevent this selection bias from occurring, an “external”, or “honest”, cross-validation process should be implemented following the feature selection at each CV stage (McLachlan, 1992; Ambroise and McLachlan, 2002; Dudoit and Fridlyand, 2003; Simon et al. 2003; Molinaro et al. 2005A; Wang et al. 2005). That is, the feature selection is performed based only on those samples set aside as training samples at each stage of the CV process, external to the test samples at each stage.

Careful consideration should be given to the feature subset selection problem when constructing a prediction rule within the framework of a supervised classification problem. This paper focuses on the implementation of external cross-validation to assess the predictive accuracy of various classification rules. Empirical results based on both univariate and multivariate feature selection procedures, for three different learning algorithms and six published microarray datasets, are presented in this paper. Of particular interest is whether the results based on the more sophisticated multivariate feature selection scheme offer a significant advantage in terms of lower error rates than results based on univariate-based feature selection.

### Datasets

The following datasets are analyzed in this paper, all of which are from Affymetrix microarrays (Affymetrix, 1999; Affymetrix, 2000a; Affymetrix, 2000b; Affymetrix, 2002). The only preprocessing that was done on each dataset was to standardize the arrays such that they each have zero mean and unit variance (an approach also used in the comparative gene expression classification study of (Dudoit et al. 2000). Standardization of microarray data in this manner achieves a location and scale normalization of the arrays. This was done to ensure that all the arrays of a given dataset were independent of the particular technology used. That is, the standardization was done to take into account the effect of processing artifacts, such as longer hybridization periods, less post-hybridization washing of the arrays, and greater laser power, to name a few. This way, for a given dataset, the values corresponding to individual genes can be compared directly from one array to another. Further, it’s been shown that this type of normalization has been effective in preventing the expression values of one array from dominating the average expression measures across arrays (Yang et al. 2001). Currently there is no universally accepted means of normalizing microarray data.

#### Colon cancer dataset (Alon et al. 1999)

This dataset consists of gene expression levels measured from Affymetrix oligonucleotide arrays (HU6000 array) for 2000 genes across 62 samples. The total intensities for the genes were obtained using the mean filtered (perfect match–mean match), or PM-MM, intensity. To compensate for variations between arrays, the intensity of each gene on an array was divided by the mean intensity of all genes on the array and multiplied by a nominal average intensity of 50. The binary classes used for analysis are normal (22 samples) and tumor (40 samples). As discussed in (Li et al. 2001), five colon samples previously identified as being contaminated were omitted (N34, N36, T30, T33, and T36), leaving the total sample size for analysis at 57. See (Alon et al. 1999) for more details on this dataset.

#### Leukemia dataset (Golub et al. 1999)

This dataset consists of gene expression levels from Affymetrix chips (HuGeneFl). The oligonucleotide arrays have 7129 probe sets over 72 samples. The binary classes used for analysis are acute myeloid leukemia (AML; 25 samples) and acute lymphoblastic leukemia (ALL; 47 samples). Intensity values were re-scaled such that overall intensities for each chip were equivalent. This re-scaling was done by fitting a linear regression model using the intensities of all genes with “P” (present) calls in both the first sample (baseline) and each of the other samples. See (Golub et al. 1999) for more details on this dataset.

#### Brain cancer dataset (Nutt et al. 2003)

This dataset consists of gene expression levels measured from Affymetrix high-density oligonucleotide chips (U95Av2) using the GeneChip software. Each array contains 12625 probe sets over 50 samples. The binary classes used for analysis are glioblastoma (28 samples) and anaplastic oligodendroglioma (22 samples). The downloaded raw expression values were previously normalized by linear scaling such that the mean array intensity for active (“present”) genes was identical for all the scans. See (Nutt et al. 2003) for more details on this dataset.

#### Brain cancer dataset (Pomeroy et al. 2002)

This dataset consists of gene expression levels measured from Affymetrix high-density oligonucleotide chips (HuGeneFl) using the GeneChip software. Each chip contains 7129 probe sets. To facilitate the binary classification framework, dataset ’A2’ from the project website was used, in which 60 medulloblastoma (MD) samples formed one class and the remaining 30 samples classified as “Other” for the second class (Note: of these 30, there were 10 malignant gliomas (MG), 10 atypical teratoid/rhaboid tumor (AT/RT), 6 supratentorial primitive neuroectodermal tumors (PNET), and 4 normal cerebellum samples). See (Pomeroy et al. 2002) for more details on this dataset.

#### Lymphoma dataset (Shipp et al. 2002)

This dataset consists of gene expression levels measured from Affymetrix chips (HuGeneFL) using the GeneChip software. Each oligonucleotide array contained 7129 probe sets over 77 samples. The two classes used for analysis are diffuse large B-cell lymphoma (DLBCL; 58 samples) and follicular lymphoma (FL; 19 samples). See (Shipp et al. 2002) for more details on this dataset.

#### Prostate cancer dataset (Singh et al. 2002)

This dataset consists of gene expression levels measured from Affymetrix chips (HU95Av2) using the GeneChip software. The number of arrays available for analysis was 102, with each containing 12600 probe sets. The two classes used for analysis are normal (50 samples) and prostate cancer (52 samples). See (Singh et al. 2002) for more details on this dataset.

## Results

### Gene Selection: Univariate vs. Multivariate

First of all, to get an idea of how effective the two GA-based feature selection processes were at selecting genes that would otherwise not be considered “top genes” from a univariate screening procedure, all three feature selection approaches were implemented in a resubstitution setting, in which all samples were used for each dataset. Table 1 provides a breakdown of the percentage of genes, relative to each gene subset size, among each of the GA-based feature selection processes that were not even among the top 100 univariately significant genes, for all six datasets.

From Table 1, one can note that for all datasets except the Golub dataset in the case of the 2-stage GA process, for gene subset sizes of 4 or more, both the single-stage and the two-stage GA approaches generated final gene subsets in which the majority of the genes of each subset size were not among the top 100 from that dataset’s univariately significant genes. This finding was especially true for subset sizes of 10, 15, 20, and 25. Whether or not this translates to much improved misclassification error rates, however, is the topic of the following two sections.

### Optimism Bias, Selection Bias, and Total Bias

Both 10-fold external and internal CV were performed, using both univariate- and GA-based feature subset selection. The idea was to consider the problem of how best to evaluate prediction rules formed from models such that the effects of optimism bias, selection bias, and “total” bias are properly taken into account, where these bias estimates are defined as follows:

(1) $$b_{ob}={MER}_{IntCV}-{MER}_{Resub}$$

(2) $$b_{sb}={MER}_{ExtCV}-{MER}_{IntCV}$$

(3) $$tb=b_{sb}+b_{ob}$$

(4) $$={MER}_{ExtCV}-{MER}_{Resub}$$

In these analyses, the empirical results were based on the same six datasets and same three learning algorithms as this study. Considering all datasets, learning algorithms, and gene subset sizes together, we found that for the results based on univariate feature selection, the average optimism, selection, and total bias estimates were only 4%, 3%, and 7%, respectively. The average optimism, selection, and total bias estimates for the GA-based results were 2%, 8%, and 10%, respectively, and those for the “GA-GA”-based results were 2%, 7.5%, and 10%, respectively. Hence, the optimism bias, incurred from using the same data to both train the classifier and estimate the classifier’s performance, from each of the GA-based analyses was half that of the univariate-based results. However, the selection bias, incurred from using the same data to both select the gene subsets and estimate the classifier’s performance, from each of the GA-based analyses was 2.5 times that of the univariate-based results.

### External Cross-Validation

**Figure 1:**
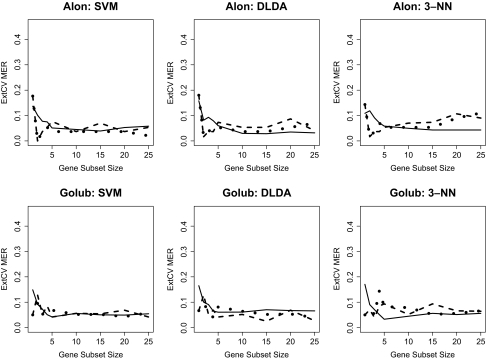
10-Fold External CV; MER vs. Gene Subset Size: Alon, Golub Datasets Solid line: Univariate FSS, Dashed line: GA FSS, Dotted line: GA-GA FSS.

**Figure 2:**
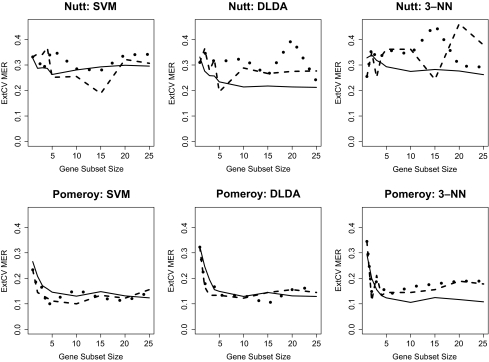
10-Fold External CV; MER vs. Gene Subset Size: Nutt, Pomeroy Datasets Solid line: Univariate FSS, Dashed line: GA FSS, Dotted line: GA-GA FSS.

**Figure 3:**
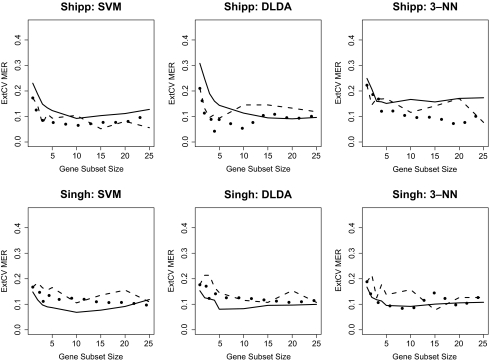
10-Fold External CV; MER vs. Gene Subset Size: Shipp, Singh Datasets Solid line: Univariate FSS, Dashed line: GA FSS, Dotted line: GA-GA FSS.

The 10-run external CV results for each dataset and classifier combination, across a number of gene subset sizes, are shown in Figures 1, 2, and 3. Within each graph, three curves are shown, corresponding to the external CV error rates based on univariate, GA, and “GA-GA” feature selection. Several observations should be noted from these Figures. First, in comparing the datasets, in general the Alon and Golub datasets had the lowest MER values for all the classifiers across the gene subset sizes, followed by the Pomeroy, Shipp, Singh, and finally Nutt datasets. Among the three learning algorithms, no single one emerged across all data-sets as the best in terms of lowest average error rates. Among the two GA-based feature selection approaches, the more complicated two-stage one did not offer a significant advantage in terms of lower average error rates over the simpler single-stage one. Further, it should be noted that for all datasets and classifiers, although the GA feature selection methods may have greater potential to select combinations of genes that are jointly discriminatory than would an approach that combines individually predictive genes, there was not a clear advantage for either of the more sophisticated GA-based methods over the univariate-based method.

**Figure 4:**
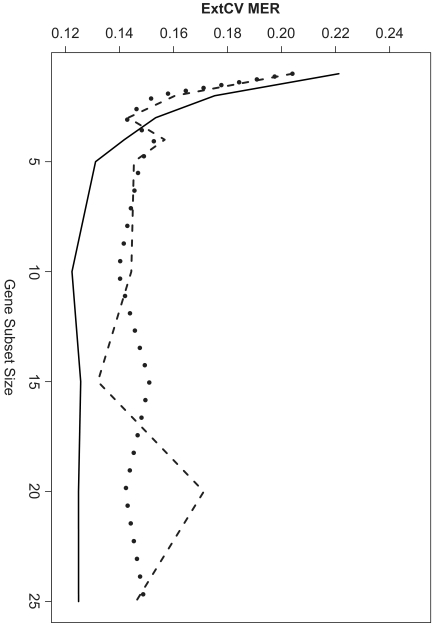
10-Fold External CV; MER vs. Gene Subset Size: All Datasets & Classifiers Together Solid line: Univariate FSS, Dashed line: GA FSS, Dotted line: GA-GA FSS.

Finally, it is interesting to consider the means and standard deviations of the 10-fold external CV misclassification error rates averaged across all datasets and classifiers, for each of the three approaches to feature subset selection. A plot of these results is shown in Figure 4. For very small subset sizes (1, 2, and 3), the GA-based methods offer a very slight advantage over the univariate feature selection approach, but beyond these sizes, the univariate method leads to lower error rates. The empirical grand means and standard deviations for each dataset across all subset sizes and classifiers, as well as the empirical grand means and standard deviations across all datasets, subset sizes, and classifiers, for each of the three feature selection methods, are available in tabular format upon request. Overall, considering all datasets, classifiers, and gene subset sizes together, the average 10-fold external CV error rates based on the univariate, GA, and GAGA feature selection approaches are all very comparable−14.2%, 14.6%, and 14.2%, respectively. It should be noted that if the Nutt data were excluded, these averages become 11.5%, 11.2%, and 10.6%, respectively.

## Discussion

An in-depth comparative study of several supervised learning methods for tumor classification based on filtered sets of genes from several published microarray datasets is provided in (Dudoit et al. 2000). The learning algorithms used in their study were linear discriminant analysis (LDA), diagonal LDA (DLDA), quadratic LDA (DQDA), classification trees, and *k*-NN. The gene selection method implemented was to select the *p* genes with largest ratio of between to within-sum-of-squares. In this study, repeated (150) runs of training/test set partitions were performed, with feature selection done only on each training set. The ratio of training to test set samples was 2:1. No cross-validation study was performed. More recently, univariate screening with both a simple *t*-test and a rank-based *t*-test (Wilcoxon Test) were used to analyze a couple of published two-class microarray datasets (Dudoit and Fridlyand, 2003). The classification schemes they used were *k*-NN, DLDA, boosting with trees, random forests, and SVM’s. In this study, they applied external and internal CV, but only using LOO CV. For both studies, the general conclusion was that the simpler classification methods such as DLDA performed better than the more complicated ones such as *k*-NN and SVM. In the more recent study, the authors found that the internal LOO CV led to misclassification error rates that were severely biased downward compared to the external CV approach.

A solid in-depth comparative study of resampling methods is provided in (Molinaro et al. 2005A). In this study, among the microarray datasets investigated were a lymphoma dataset (Rosenwald et al. 2002) and a lung tumor dataset (Bhattacharjee et al. 2001), both of which were two-class datasets, marked by small sample sizes relative to extremely large numbers of genes. The authors implemented LDA, DLDA, *k*-NN, and classification and regression trees (CART) as their learning algorithms. The gene selection method used was univariate in nature (*t*-tests). To assess the predictive ability of the prediction models built, the authors used *K*-fold CV, LOO CV, Monte Carlo CV (MCCV), and .632 + Boostrap (Efron, 1983; Efron and Tibshirani, 1993). For both microarray studies, the authors found that .632+, LOO CV, and 5- and 10-fold CV had the smallest MSE and bias. More detailed results for the lung tumor study are provided in (Molinaro et al. 2005B). The authors also found that 10-fold CV prediction error estimates approximated the results of LOO CV in almost all analyses, which was promising considering the increased computational burden of LOO CV over 10-fold CV. Furthermore, they confirmed the importance of using both repeated-run 10-fold CV, in terms of lowering the MSE, bias, and variance of standard 10-fold CV. Finally, the authors concluded that when honest CV was implemented in small-sample settings with large-to-intermediate signal-to-noise ratio (e.g., microarray data), both LOO CV and 10-fold CV outperformed the .632 + bootstrap.

In another study (Xiong et al. 2001), there were two multivariate feature selection methods used –a Monte Carlo method and a stepwise forward selection method. Three binary classification data-sets were used in this study: The results are based only on using Fisher’s LDA as the classification mechanism. Also, these authors used a “holdout” method to evaluate the performance of the selected genes, dividing the data into a training and test set in the following proportions: (50%, 50%), (68% and 32%), and (95% and 5%), respectively, and then averaged the results of 200 runs of each of these approaches. In this study, it was found that both multivariate methods performed better than the univariate-based *T*-test and prediction strength statistic methods (Golub et al. 1999). However, the accuracy of classification criterion for forming gene subsets was based on the total collection of tissue samples, which allows for the presence of selection bias. In addition, the only subset sizes considered in this study were 1, 2, and 3.

External CV was implemented on two published datasets (Ambroise and McLachlan, 2002). The samples were randomly divided into 50 different training and test set partitions, with the CV performed only on the training data. They used two schemes for multivariate feature selection and classification–backward selection with SVM and forward selection with LDA. No univariate-based approach to perform the feature selection was implemented. They considered the effect of selection bias by performing external 10-fold CV and internal LOO CV. Unfortunately, no internal 10-fold and external LOO results were provided in the study. The average values of the error rate estimates across the multiple runs were obtained for both approaches for each dataset. They found that the internal LOO CV led to overly optimistic error rates compared to the external 10-fold CV process, for both classification schemes and datasets.

With respect to GA’s, there has been some work with them in the context of classification of microarray data. In (Li et al. 2001), a GA was implemented on a training set from the colon cancer data from (Alon et al. 1999) to select a number of 50-gene subsets that discriminate between normal and tumor tissue samples. 3-NN was used as the objective function within the GA. Once a large enough number (6348) of these 50-gene solutions were obtained, the solutions were pooled together such that a frequency count could be performed. That is, using all the genes comprising these solutions, a ranked list of the most often selected genes was formed. From this list, the top D genes were used to classify test set samples using the 3-NN classifier. The authors found that the test set predictions stabilized when as few as 25 and up to 110 top genes were used. As more top genes were included, the number of unclassifiable samples increased. The same GA/*k*-NN method was used for training of 38 samples from the leukemia dataset from (Nutt et al. 2003). Using the top 50 most frequently selected genes among the 50-gene subsets generated by the GA/3-NN method, they correctly classified 33 of the 34 test samples.

Although applied to a different type of micro-array platform, in a multiclass cancer classification setting (as opposed to a two-class setting as has been the focus in the current study), a more recent study that incorporates the GA as the feature selection algorithm is (Liu et al. 2005). Here, the authors used a GA/SVM method to perform multiclass cancer categorization on two spotted cDNA datasets –the NCI60 9-class dataset (Ross et al. 2000) and the Brown 14-class dataset (Munagala et al. 2004) –both of which were marked by small sample sizes relative to very high feature sizes. To assess the predictive accuracy of their classifiers, the authors implemented only LOO CV. However, there was no clear mention of whether external (honest) CV was used. The authors found that 40 genes were sufficient to allow highly accurate multiclass tumor distinctions, and SVM was the technique of choice for potentially noisy and sparse data such as the data they had. No univariate feature selection method was compared in this study, but the potential advantage of GA’s over univariate rank-based feature selection techniques was discussed.

Finally, the GA was also implemented as part of a comparative study in (Wang et al. 2006). Here, the authors investigated the merging of microarray data across institutions and microarray platforms. The two datasets that were combined were the Harvard Singh prostate cancer (Affymetrix) dataset (Singh et al. 2002) and the Harvard Lapointe prostate cancer (cDNA) dataset (Lapointe et al. 2004) –both of which were marked by very high numbers of features relative to much smaller numbers of samples. Their prediction models included logistic regression with forward stepwise feature selection, and LDA combined with several multivariate feature selection algorithms -- their own robust greedy feature selection (RGFS) algorithm, with principal components analysis (PCA) feature selection, with a greedy algorithm similar to stepwise forward selection, and with a GA using mahalanobis distance as the fitness function. No univariate feature selection method was compared in this study. LOO CV was the algorithm used to assess the predictive accuracy of their models. The authors also incorporated the feature selection processes into the CV loops, and in the process stressed the importance of performing this type of honest CV when assessing predictive models. They found that the LOO CV misclassification error rates were generally to the order of 25—30%, and overall that the problem of finding a definitive method for performing feature selection is far from finished.

The current research builds on the findings of the studies of (Ambroise and McLachlan, 2002), (Xiong et al. 2001), (Dudoit and Fridlyand, 2003), (Molinaro et al. 2005A), and (Wang et al. 2006), in the sense that 10-fold external CV was implemented to take into account selection bias when estimating the misclassification error of a classification rule based on microarray data. However, in this research, the external CV is performed in conjunction with both univariate- and multivariate GA-based feature selection to assess the performance of various prediction rules across six two-class microarray datasets. The current research also extends on the GA-based analyses of (Li et al. 2001) and (Liu et al. 2005), in that the GA is actually incorporated into each stage of a 10-fold (external) CV procedure, rather than have the data split into a training and test set. It also builds on the results of (Li et al. 2001) in that once subsets of genes are selected by the GA (single-stage approach), they are not then re-pooled together such that the final gene subsets used for modeling are actually selected from a new pool of genes based on frequency of selection among the final gene subsets selected by the GA procedure–ultimately an inherently univariate notion of feature selection. Instead, in this research the GA-selected gene subsets are left alone and not broken-up and regenerated based on frequency of selection. The truly best solution as selected from the GA is preserved for use in the classification algorithm. Also, a simpler and less computationally intensive fitness function than *k*-NN, Mahalanobis distance, is employed in the GA algorithm implemented in this research. More details on the GA methods used in this study are provided in the Methods Section.

Repeated- (10-) run 10-fold external cross-validation was applied to each of six datasets, using each of three different learning algorithms. With the external CV approach, the feature selection was performed at each stage of the CV process, based only on the training set partitions of each stage and hence external to the test sets used to evaluate the models. The average error rates across all the classifiers and gene set sizes were very comparable among the univariate and two multivariate feature selection approaches, as they were all 14%. In terms of datasets, only the Nutt dataset had noticeably higher error rates across classifiers and subset sizes than those of the other datasets, as they were, on average, above 27% for each of the three feature selection approaches. Ultimately, the misclassification rates did not vary significantly by dataset, for five of the six datasets at least, suggesting that these results should generalize well to other clinical microarray datasets. The same generalization ability should hold with respect to classifiers, since the three classifiers used function in different ways, and since there is no clear reason to suspect that the results are connected to the method of classification.

## Conclusions

For each of the six datasets used in this study, we have shown that although a multivariate-based approach to feature subset selection may have greater potential to select combinations of genes that are jointly discriminatory than would a method that combines individually predictive genes, there was no clear advantage of the more computationally intensive GA approaches over the simpler univariate ones in estimating the prediction error for a classification rule constructed from a selected subset of genes from microarrays. Considering all classifiers, subset sizes, and learning algorithms, we found that the optimism bias estimates from the GA analyses were half that of the univariate approach, while the selection bias estimates from the GA analyses were 2.5 times that of the univariate results. This higher selection bias suggests that selecting genes in multivariate models using a GA may be more likely to select spurious genes than would be the case with a univariate-based approach. This finding makes sense in that since the selection bias measures the bias in the estimate of CV prediction error due to feature selection, one would suspect that it would be higher with the multivariate feature selection approach since this approach searches a much higher dimensional model space when finding the features. Thus, with the multivariate feature selection approach, it would naturally be more possible to include spurious genes in candidate models, which can be seen as overfitting the data. That is, considering the notion of overfitting to mean that too much flexibility is allowed in the model space, such that the models trace the data too closely, likely select spurious features of the given data set, and hence do not accurately generalize to independent validation data, it would be safe to say that the GA-based methods tend to overfit the data. Ultimately, whether a univariate or a GA-based feature selection approach is used, the presence of optimism and selection bias should be taken into account through the use of external CV.

## Methods

### Supervised learning methods

In this study, three well known and widely used choices of supervised learning algorithms were implemented: support vector machines (SVM’s), DLDA, and *k*-NN (*k*=3 in this study). For more details on each of these classifiers, the reader should refer to (Dudoit and Fridlyand, 2003) and (Dudoit et al. 2000).

### Feature subset selection

#### Univariate Feature Selection

A univariate-based means of feature subset selection was used to perform gene selection. Rank-based, unequal variance *T*-tests were performed on each of the genes from the designated training sets of samples among each of the six datasets. In each training set, this resulted in an ordered list of “top genes”. This list, ordered according to increasing *p*-value, was then used in generating various “top gene subset size” models. To obtain a Monte Carlo type of estimate of the 10-fold external CV misclassification error rates, the standard 10-fold process is run 10 separate times, and the average of the resulting ten 10-fold CV MER estimates is recorded. For a given dataset, for each of the classifiers implemented for a given dataset, the same ten training and test set partitions for a given iteration were used to maintain consistency in interpreting the repeated-run 10-fold CV results.

#### GA and “GA-GA” Feature Selection

A multivariate-based means of feature subset selection, the genetic algorithm (GA), was used to perform gene selection. For more details on the GA in general, the reader is referred to (Holland, 1975; Mitchell, 1997; Freitas, 2001). Both a single-stage and a two-stage GA feature selection process were implemented. Repeated (10) runs of the GA process are used to provide more stable estimates of the 10-fold CV error rates. The repeated runs are implemented via the genalg software, which provides the user with the opportunity to run the entire GA multiple (10) times for each training set of data (in the case of external CV, the GA is applied 10 times on each of the 10 training subsets of samples, corresponding to each stage of the 10-fold CV process). It should be noted that to evaluate each candidate *d*-gene subset, Mahalanobis distance is used as the fitness function for sample classification based on the *d* genes. This metric was chosen as the fitness function based on its lighter computational burden than a more complicated algorithm such as *k*-NN. For more information on the genalg software, the reader should refer to (Baggerly et al. 2003).

**Figure 5:**
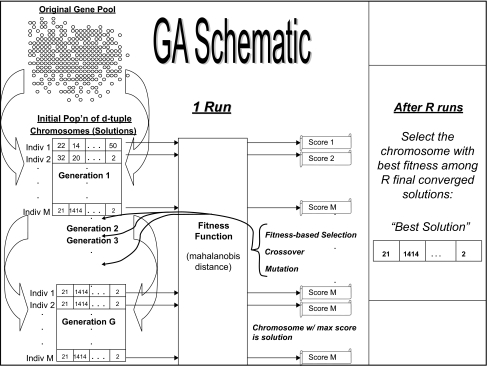
Schematic of 1 Run of Basic GA Process

For the single-stage GA approach, the GA considers all *p* genes of each dataset. The number of *d*-gene solutions (“chromosomes”), *M*, selected by each implementation of the single-stage GA is 1000, so over *R* = 10 iterations, a “superpopulation” of 10000 candidate solutions are obtained. The number of generations, *G*, to run for each iteration of the GA was set to 250, which we found was large enough to ensure convergence of the 1000 solutions. Of the 10000 solutions, there will be as many as 10 unique solutions that will have converged after 250 generations, corresponding to each of the 10 runs of the GA). Of these 10 converged solutions, the one with the best fitness score is retained for use in the classification algorithm, which in-turn preserved the actual solutions discovered by the GA (as opposed to breaking-up the discovered solutions based on frequency of selection of each individual gene in the chromosomes, as was done in (Li et al. 2001)). Note that for the external 10-fold CV procedure, this process is applied within each of the 10 stages of the 10-fold CV. A schematic of one run of the basic GA process just described is provided in Figure 5.

For the two-stage approach (“GA-GA”), the first stage GA begins with all *p* genes, as done in the standard GA process just described. However, for the second GA stage, the algorithm is applied to a reduced initial gene pool, based on the initial GA’s selection results. This new gene pool is composed of the union of all genes selected among the final generation’s population of 1000 *d*-gene solutions from the first stage of GA, for all 10 iterations of the GA. That is, the second stage’s GA procedure uses as its initial gene pool all genes that appeared at least once among the “superpopulation” of 10000 solutions obtained from the initial GA stage. This way, genes that may appear in just a small proportion of the 1000 final solutions of any given iteration, but still appear in multiple iterations of the GA, now have a better chance of being considered for use in building classifiers. Thus, the idea behind the second implementation of the GA would be to attempt to select the ’best of the best’ genes from a given training dataset. It should be noted that for the second stage GA, the number of generations remained at 250, but the number of *d*-gene solutions selected by each implementation of the GA was reduced to 500, since the initial gene pool was reduced considerably. Finally, note again that for the external 10-fold CV procedure, this process is again applied within each of the 10 stages of the 10-fold CV.

## Figures and Tables

**Table 1 -:** % of genes of each subset size not within top 100 univariately significant genes list.

	Alon		Golub		Nutt		Pomeroy		Shipp		Singh	

Size	GA	GAGA	GA	GAGA	GA	GAGA	GA	GAGA	GA	GAGA	GA	GAGA
1	0.0	0.0	0.0	0.0	0.0	0.0	0.0	0.0	0.0	0.0	0.0	0.0
2	0.0	0.0	0.0	0.0	0.0	0.0	0.0	0.0	50.0	0.0	0.0	0.0
3	66.7	66.7	33.3	33.3	33.3	66.7	33.3	100.0	100.0	66.7	0.0	33.3
4	50.0	50.0	50.0	25.0	75.0	75.0	50.0	50.0	75.0	100.0	50.0	50.0
5	20.0	60.0	40.0	20.0	100.0	80.0	80.0	60.0	60.0	80.0	60.0	60.0
10	70.0	70.0	60.0	60.0	70.0	90.0	70.0	80.0	70.0	80.0	60.0	60.0
15	73.3	66.7	60.0	80.0	93.3	86.7	66.7	60.0	93.3	80.0	73.3	73.3
20	75.0	75.0	70.0	80.0	95.0	95.0	85.0	80.0	85.0	85.0	85.0	95.0
25	84.0	84.0	76.0	72.0	96.0	92.0	76.0	84.0	96.0	96.0	84.0	76.0
